# Gestational Trophoblastic Disease: Diagnostic and Therapeutic Updates in Light of Recent Evidence: A Literature Review

**DOI:** 10.3390/medicina61091642

**Published:** 2025-09-10

**Authors:** Giuseppe Gullo, Marinì Satullo, Eleonora Conti, Silvia Ganduscio, Elena Chitoran, Zoltan Kozinszky, Karolina Kowalcze, Robert Krysiak, Valentina Billone, Gaspare Cucinella

**Affiliations:** 1 Department of Obstetrics and Gynaecology, AOOR Villa Sofia–Cervello, University of Palermo, 90100 Palermo, Italy; marinisat@gmail.com (M.S.); elexconti97@gmail.com (E.C.); gandusciosilvia@gmail.com (S.G.); valentina.billone@gmail.com (V.B.); gasparecucinella1@gmail.com (G.C.); 2 General Surgery and Surgical Oncology Department I, Bucharest Institute of Oncology, 022328 Bucharest, Romania; 3 Department of Obstetrics and Gynecology, University of Szeged, 6725 Szeged, Hungary; kozinszky@gmail.com; 4 Capio Specialized Center for Gynecology, Solna, 182 88 Stockholm, Sweden; 5 Department of Pathophysiology, Faculty of Medicine, Academy of Silesia, Rolna 43, 40-555 Katowice, Poland; kkowalcze@sum.edu.pl; 6 Department of Pediatrics in Bytom, Faculty of Health Sciences in Katowice, Medical University of Silesia, Stefana Batorego 15, 41-902 Bytom, Poland; 7 Department of Internal Medicine and Clinical Pharmacology, Medical University of Silesia, Medyków 18, 40-752 Katowice, Poland; rkrysiak@sum.edu.pl

**Keywords:** trophoblastic disease, hydatidiform mole, gestational trophoblastic neoplasia

## Abstract

*Background/objectives*: Gestational trophoblastic diseases (GTDs) are rare premalignant and malignant conditions characterized by abnormal proliferation of trophoblastic tissue. They are often asymptomatic but may present with vaginal bleeding. GTDs include hydatidiform moles and gestational trophoblastic neoplasms (GTNs). Current research aims to improve diagnostic tools and treatment strategies to reduce cancer risk and improve survival. Increasing attention is being paid to immunotherapy and treatment personalization, with the goal of minimizing long-term side effects and enhancing quality of life. Less toxic therapies are ideal for low-risk patients to reduce drug-related toxicity. *Materials and Methods:* A narrative review was conducted to analyze studies from the last twenty years on the diagnosis, staging, and treatment of GTDs. Sources included PubMed, Scopus, and Cochrane Library, using keywords such as “trophoblastic disease,” “hydatidiform mole,” and “gestational trophoblastic neoplasia.” *Results:* In recent years, the clinical management of gestational trophoblastic disease (GTD) has made significant progress through diagnostic, prognostic, and therapeutic innovations. More sensitive imaging techniques and serial monitoring of serum β-hCG now allow early diagnosis of hydatidiform mole and gestational trophoblastic neoplasia (GTN), reducing the risk of complications and metastasis. *Conclusions:* In the last decade, GTD management has improved significantly, with better diagnostic techniques, standardized staging, and more effective treatments. However, challenges persist, including relapse management, long-term monitoring, and psychological support. Early diagnosis is key, with ultrasound being essential for detecting abnormalities in the first weeks of pregnancy. Staging follows FIGO and WHO criteria, considering hCG levels and metastasis. This review highlights recent advances in diagnostic tools, emerging therapies—including immunotherapy—and the need for personalized, less toxic treatment approaches to improve patient outcomes.

## 1. Introduction

Gestational trophoblastic diseases (GTDs) are rare pathological conditions originating from the placenta and are characterized by abnormal proliferation of trophoblastic tissue. They are a spectrum of diseases: in fact, GTDs include hydatidiform moles and gestational trophoblastic neoplasms (GTNs).

Before the advent of sensitive assays for human chorionic gonadotropin (hCG) and effective chemotherapy, there was a high mortality rate for malignant variants of this insidious disease. Nowadays, a proper approach to this condition allows women to be offered effective treatment and preserve their future fertility, but early and timely management of this condition and appropriate follow-up is important [1].

The epidemiology of these diseases varies considerably depending on geographic area, maternal age, and other risk factors.

In Western countries, the incidence of a complete hydatidiform mole, which is the most common type within the spectrum of GTD, is around one case per 1000–2000 pregnancies, while partial mola is slightly more frequent, with about three cases per 1000 pregnancies. Malignant variants of the disease, such as choriocarcinoma and trophoblastic placental site tumors, are fortunately rarer, with estimates ranging from one case per 20,000 to 50,000 pregnancies. The scenario is different in developing countries where the incidence can be up to two to three times higher than in industrialized countries. The reasons for this epidemiological diversity have not yet been fully elucidated, but it is possible that nutritional and genetic factors may significantly impact it [2,3,4,5].

Another element not to be underestimated concerning the incidence of these pathological conditions is maternal age. Women with a higher risk of developing a hydatidiform mole are either very young (<16 years) or elderly (>40 years), with a peak incidence for complete mola over 45 years. A positive history of a previous GTD is also a serious risk factor: after a first episode of mola, the risk of recurrence increases to 1–2% in subsequent pregnancies, compared with a baseline risk of about 0.1% [6].

GTD is thus considered a rare but not negligible condition with a heterogeneous geographic distribution.

Clinically, these pathologic variants share often similar symptoms such as vaginal bleeding, pelvic pain iperemesis, anemia, and thyrotoxicosis; however, histologically, GTD is divided into hydatidiform disease (containing villi) and other trophoblastic tumors (without the presence of villi) [7].

A hydatidiform mole (HM) can be classified as complete or partial mola and is usually considered the noninvasive form of GTD [7,8,9]. Although HMs are generally thought to be benign, they are indeed premalignant forms and can potentially evolve into a malignant and invasive condition [10,11]. Malignant variants of GTD are termed gestational trophoblastic neoplasms (GTNs) and include invasive moles, choriocarcinoma, the epithelioid trophoblastic tumor (ETT), and the placental site trophoblastic tumor (PSTT) [7].

GTN can give rise to metastasis and is a life-threatening condition if not properly treated. An invasive mole is described when abnormal trophoblastic cells penetrate deeply into the uterine wall, resulting in complications. The rapid growth of abnormal tissue can result in abnormal uterine bleeding [12].

Choriocarcinoma is a rare and aggressive neoplasm. There are two main subtypes of choriocarcinoma: gestational and nongestational. Choriocarcinoma is predominantly female pathology, but it can also develop in men, usually as part of a mixed germ cell tumor [7].

ETT is a rare type of GTN that derives from trophoblastic cells that exhibit an epithelioid cell morphology; they can behave as an aggressive tumor. ETT may manifest with abnormal vaginal bleeding or the presence of a mass in the uterine cavity [8]. PSTT is another form of GTN, also rare, which originates from the placental implantation site. Unlike the more frequent trophoblastic tumors, PSTT tends to give manifestation of itself months or years after a canonical pregnancy; it is a tumor characterized by slow growth and may give rise to irregular vaginal bleeding [8,13]. Crucial, as already mentioned, for these diseases is early diagnosis and careful follow-up. An important resource is imaging, especially ultrasonography. It is also important to take advantage of the aid of histological analysis. Treatment can be a challenge with regard to fertility preservation, chemotherapy regimens, and subsequent monitoring [7].

## 2. Materials and Methods

A narrative review of the scientific literature was conducted using the PubMed, Scopus, and Cochrane Library databases with a focus on studies published in the last twenty years. The search was conducted using the keywords “trophoblastic disease,” “hydatidiform mole,” and “gestational trophoblastic neoplasia.” This initial search returned 2205 articles. After applying filters to include only English-language publications involving human adults (female, aged 19 years or older), the number of relevant studies was narrowed to 650. Data were extracted from these articles, and additional sources were identified through manual review of references cited in the selected articles.

## 3. Results

### 3.1. Hydatidiform Mole

A hydatiform mole (HM) is defined as an unusual and altered proliferation of the trophoblast and the placental tissue, caused by an abnormality in fertilization and divided into two different types: complete and partial. It is considered a benign condition, but with evolutionary potential toward malignancy [14]. Hydatidiform moles are typically diagnosed during the first half of pregnancy. The most frequent characteristic symptom is abnormal bleeding. Other frequent signs and symptoms include increased uterine volume greater than expected for the gestational age, absence of fetal heartbeat, cystic enlargement of the ovaries, hyperemesis, and an abnormally elevated hCG level that does not match the canonical level for gestational age [1]. A complete mole is a pathological variant in which all genetic material is of paternal origin. In about 90% of cases, it occurs in the situation in which an anucleated oocyte, i.e., lacking maternal genetic material, is fertilized by a single spermatozoon, which then duplicates its own genetic makeup, resulting in a 46,XX karyotype; in about 10% of all cases, complete mola may originate from the fertilization of an oocyte lacking maternal DNA by two spermatozoa, resulting in the formation of a 46,XY karyotype. In both cases this situation is distinguished by the absence of any maternal genetic contribution. In fact, this process does not lead to embryonic development, and there is widespread trophoblastic proliferation with the typical cystic vesicles devoid of fetal elements that are recognized on imaging [15]. A partial mole is distinguished genetically by being a triploidy: the chromosome set consists of as many as 69 chromosomes. The most frequent karyotype is 69,XXY, followed by 69,XXX, and, rarely, 69,XYY. Partial mola originate in most cases when two sperm fertilize a single oocyte. In partial mola, therefore, there is a maternal genetic contribution, and often the development of an embryo or fetus with abnormal and nonviable characteristics is described [16]. Nowadays, to make a diagnosis of hydatidiform mola, ultrasonographic examination is essential. Molar tissue is recognized by the presence of a mixed echogenic pattern that replaces the placenta, produced by villi and intrauterine blood clots, but these findings may be of doubtful interpretation because they are poorly represented or absent in cases of early complete moles and partial moles [1].

The most evocative ultrasound images are represented, in complete mola, by a typical appearance called “snowstorm” or “grape cluster” due to the presence of cystic chorionic villi and the absence of the embryo/fetus. In partial mola, a fetus can be observed, but in most cases with structural malformations, along with disorganized placenta and cystic areas, placentomegaly with “Swiss cheese” appearance, and an irregular gestational sac. [17,18]. The most frequent clinical presentation of hydatidiform mola is often characterized by the following signs and symptoms: abnormal vaginal bleeding, uterus bulkier than gestational age, absence of fetal heartbeat, hyperemesis gravidarum, and signs of hyperthyroidism [12]. In addition to the ultrasound criterion, a serum β-hCG assay is unavoidable. In complete mola, particularly high values (>100,000 mIU/mL) are observed, exaggerated relative to gestational age. High levels always occur in partial mola but are usually lower than in the complete form. High β-hCG levels are often associated with an increased risk of complications, such as progression to malignant variants [19]. The association of evocative ultrasound images with an increase in β-hCG above that expected for gestational age is highly suggestive of molar pregnancy [1]. Thanks to the routine use of ultrasonography and with sophisticated software and the advent of much more hCG-sensitive tests than in the past, hydatidiform moles can now be diagnosed very early and, as a result, the classic features of hydatidiform moles are observed less often than in the past [20,21].

Definitive diagnosis can clearly be obtained only by histologic examination where we observe, in complete mola, absence of embryonic tissue and diffuse hydropic villi with marked trophoblastic atypia and diffuse distribution and, in the partial mola, characteristic histologic features are presence of fetus or embryonic tissue and focal hydropic villi with mild to moderate atypia, typically with a mosaic pattern [22].

Several markers have been studied in relation to HM; for example, a study using a sample of 237 patients with HM demonstrated a significant reduction in the expression of the anti-apoptotic marker BCL-2 in the complete versus partial mola, suggesting increased uncontrolled proliferation in the complete variant [23].

Diagnosis of hydatidiform mola often occurs incidentally after histologic examination of material taken following a revision of uterine cavity performed in a suspected miscarriage. Following this procedure, patients are monitored with serial hCG assays. In case of early suspicion of hydatiform mola, pathological material should be evacuated as soon as possible. Uterine cavity revision (dilatation, evacuation, aspiration) is the most commonly used method in such cases, usually performed under ultrasound guidance [1,19,24].

In the case of maternal Rh-negativity, prophylaxis with anti-D immunoglobulin is recommended following evacuation [25].

At the time evacuation occurs, serum β-hCG should be assayed to define the starting value from which seriate monitoring begins [26].

In patients with complete HM, it is important to measure β-hCG levels weekly until three consecutive negative values (<5 mIU/mL) are obtained; once this has been achieved, monthly checks of this value are performed for a total of six months from the first negative result [27]. In complete molar pregnancy, a monthly blood sample is taken to measure β-hCG for 6 months after the first normal value [26].

In partial HM, in weekly dosing, two consecutive normal values are required instead of three. After that result, dosing is performed one month after the first normal value: if there is no increment in the studied value, follow-up can be stopped [24].

A study enrolling 4 701 patients showed that for 98% of cases of malignant trophoblast pathology, the condition manifested within six months of the β-hCG value returning to the normal range; this confirmed the modus operandi whereby follow-up is discontinued after a six-month time frame [28].

GTN is suspected if the β-hCG remains stable with <10% variability after 3–4 measurements, or if it resumes an increase considering three consecutive assays, or again, if it remains positive over a period of more than 6 months [26].

Although mola evacuation with dilatation and ultrasound-guided aspiration is the method of choice, if the woman does not desire offspring or is >40 years old, a hysterectomy may be proposed [29].

Hysterectomy in the therapeutic management of hydatidiform mola is not a routine approach but can be applied in circumscribed cases with precise characteristics: age over 40 years with completed reproductive desire since complete mola in women of greater age has an increased risk of evolution into GTN, in the face of uncontrollable massive uterine hemorrhage where hysterectomy may prove to be a life-saving procedure, or even in cases of recurrence or persistence post evacuation. Also in the case of concomitant gynecologic diseases such as severe endometriosis, multiple fibromatosis, or carcinomas, hysterectomy might be considered. Even following hysterectomy, follow-up with β-hCG is mandatory since trophoblastic disease can metastasize and a GTN can arise even in the absence of a uterus [30].

If hysterectomy is opted for, adnexa are preserved in most cases. A hysterectomy reduces the risk of postmolar malignant evolution to about 3–5%, compared with about 15–20% after evacuation by dilatation and aspiration [31].

Although a very rare condition, coexistence of a fetus with a molar alteration of the placenta is possible. This situation occurs in approximately 1 in 22,000–100,000 pregnancies [32,33].

In fact, the literature on the subject consists almost exclusively of limited reviews and single case reports. Cases of both partial and complete hydatiform mola with a coexisting normal fetus have been described. Often the diagnosis of such rare and complex situations is early, due to the ultrasound finding of a cystic placental portion distinct from the fetoplacental unit, but, more infrequently, the diagnosis is not suspected until placental histologic examination after delivery. Frequent in this condition are complications such as hemorrhage or gestational hypertension that require termination of pregnancy, but cases are described of patients who have given birth to live, viable infants. No major congenital abnormalities were reported in the children who were born [33,34].

It is important to emphasize that a hydatidiform mole can be differentially diagnosed with various conditions affecting the first trimester of pregnancy, such as miscarriage and ectopic pregnancy. Indeed, the three conditions share similar clinical features, making differential diagnosis sometimes challenging. However, a correct diagnosis is essential to identify appropriate treatment and prevent complications. In all three medical conditions, bleeding from the external genitalia is a common symptom. However, in hydatidiform moles, this is often accompanied by a larger uterus and, in some cases, vomiting. In cases of miscarriage, vaginal bleeding is often associated with abdominal cramps and expelled embryonic tissue. In ectopic pregnancy, abdominal pain can vary in intensity. Ultrasound and β-hCG monitoring are crucial in the differential diagnosis. Ultrasound may reveal the typical snowstorm-like appearance of a hydatidiform mole. In cases of miscarriage, possible embryonic remnants may be detected. In cases of ectopic pregnancy, signs of an adnexal mass or an ectopic gestational sac may be suggestive. β-hCG values are very high in the case of a hydatidiform mole (>100,000 IU/L); in spontaneous abortion, values generally progressively decrease, and ectopic pregnancy is characterized by a slower growth of β-hCG values and often a plateau is observed [12].

### 3.2. Postmolar Gestational Trophoblastic Neoplasia

Postmolar gestational trophoblastic neoplasia is a pathologic condition that can occur following evacuation of the hydatidiform mole when β-hCG levels have an increase or remain unchanged; early diagnosis of the situation and appropriate intervention are essential in these cases [1].

We can describe the diagnostic criteria for postmolar gestational trophoblastic neoplasm. According to FIGO guidelines, one or more of these elements is required to raise suspicion of this complication [35]:

- β-hCG plateau: at least four consecutive values (days 1, 7, 14, 21) with ≤10% change;
- β-hCG increase: ≥10% increase over three consecutive values (days 1, 7, 14);
- Persistence of β-hCG beyond 6 months after molar evacuation;
- Positive histology for choriocarcinoma regardless of β-hCG trend;
- Presence of metastatic disease (e.g., lung, brain, liver, GI) with positive β-hCG.

Other non-diagnostic but supportive criteria for recognition of pathology are presence of metastasis, persistent vaginal bleeding after evacuation, increased uterus volume, and systemic symptoms such as headache, cough, and dyspnea [36,37].

The role of repeat uterine cavity revision in increasing or stabilizing β-hCG levels is debated. Some studies show that repeating curettage promotes remission in less than 20 percent of cases, with uterine perforation occurring in 4–8 percent of patients [38,39].

In contrast, Pezeshki et al. report that 68 percent of 544 patients studied achieved remission after the second uterine cavity revision, without complications such as post perforation hysterectomy [40].

Beyond the controversial and debated second cavity revision, the therapy of choice in these cases is a single-drug or multi-drug approach, depending on risk [41].

The basic treatment in these cases is based on the use of single-drug chemotherapy with methotrexate (MTX) plus folinic acid or actinomycin D (ActD). A study of 150 patients with low-risk post-molar GTN revealed that a high β-hCG value one week after the first course of MTX predicts the need for additional courses or a change in therapy [42].

The 2017 SEOM guidelines state to use the MTX/FA combination as the first choice, continuing therapy for 6 weeks after hCG negativization [43].

In high-risk cases, multi-drug chemotherapy with the EMA-CO protocol (etoposide, MTX, ActD, cyclophosphamide, vincristine) is suggested, with about 78% complete remission [44,45,46].

As already mentioned, the 2023 systematic review and meta-analysis by Albright et al. examines the treatments used in high-risk gestational trophoblastic neoplasia, providing results on clinical outcomes, response, survival, and toxicity. This is a review of studies published between 2000 and 2021 in which an analysis of chemotherapy regimens was conducted, in particular EMA-CO (etoposide, methotrexate, actinomycin D/ cyclophosphamide, vincristine), comparing it with other protocols. This meta-analysis showed that EMA/CO is the most widely used drug protocol, with a complete response rate of 72–91% and overall survival of up to 94% in some cohorts. Furthermore, the results of this important study highlight that EMA-CO is usually well tolerated, although it can sometimes cause adverse effects such as neuropathy (particularly due to vincristine), myelosuppression, and risks to fertility [46].

EMA-CO has also been shown to be effective in cases of metastatic disease: a study of 30 patients with metastases documented 93% survival and 67% permanent clinical response [47]. The EMA-CO protocol is usually fairly well tolerated, with severe hematologic toxicity in about 13–32% of cycles and serious long-term effects such as secondary leukemias and cancers in 3–4% of patients [1].

### 3.3. Gestational Trophoblastic Neoplasia

Gestational trophoblastic neoplasia (GTN) represents a rare group of malignant disorders arising from abnormal proliferation of trophoblastic tissue following conception. The main subtypes include invasive mola, choriocarcinoma, the placental site trophoblastic tumor (PSTT), and the epithelioid trophoblastic tumor (ETT) [48].

An invasive mole typically arises from complete hydatidiform moles, although it may also follow partial moles. Histologically, it is characterized by edematous chorionic villi with trophoblastic proliferation invading the myometrium, which distinguishes it from choriocarcinoma [49]. Metastases occur in only about 4% of cases, with the lungs being the most common site (80%), followed by the vagina (30%), pelvis (20%), liver (10%), brain (10%), bowel, kidneys, and spleen [50]; ovarian metastases are very rare. Definitive diagnosis would ideally be histological following hysterectomy, but since— as mentioned— hysterectomy is not commonly performed, diagnosis is often made based on β-hCG levels and radiologic imaging, and chemotherapy is usually initiated before histological confirmation is obtained [51].

Choriocarcinoma is the most common malignant form of gestational trophoblastic neoplasia (GTN). Approximately 50% of gestational choriocarcinomas arise after a molar pregnancy, while 25% occur following a term pregnancy, and the remaining 25% develop after other gestational events, such as miscarriage or ectopic pregnancy [52]. It is the most aggressive and highly vascular form of GTN, characterized histologically by a biphasic proliferation of cytotrophoblast and syncytiotrophoblast without chorionic villi. It often presents with rapid hematogenous dissemination, particularly to the lungs, brain, and liver [53].

PSTT is a rare variant of GTN (<2% of cases) arising from intermediate trophoblasts at the placental implantation site, with myometrial invasion occurring in approximately 50% of cases. As with choriocarcinoma, chorionic villi are absent. In contrast to choriocarcinoma, PSTT tends to grow slowly, is less sensitive to chemotherapy, and is associated with lower β-hCG levels, which may complicate diagnosis [54].

ETT is another rare GTN subtype (<1%), originating from a chorionic-type intermediate trophoblast. In most cases, it develops in the cervix or lower uterine segment. It often presents years after a term pregnancy and can be mistaken for PSTT or even squamous cell carcinoma of the cervix due to its histological features [55]. ETT typically presents with mildly elevated β-hCG levels.

Clinically, GTNs present with a wide range of symptoms, depending on the antecedent pregnancy, the type of disease, and its extent. The most common symptom is abnormal vaginal bleeding. Choriocarcinoma often presents with dyspnea, cough, chest pain, tachypnea, and hemoptysis, due to pulmonary metastases and the embolic potential of trophoblastic tissue. Liver metastases, though rare, may lead to intra-abdominal bleeding. Brain metastases can be asymptomatic or cause symptoms ranging from mild headaches to severe complications such as intracranial hemorrhage [51]. PSTT and ETT, as previously mentioned, typically present with irregular bleeding some time after the preceding pregnancy. Additionally, rare symptoms such as virilization and nephrotic syndrome have also been reported [56].

β-hCG plays a fundamental role in the diagnosis of GTN, with levels often exceeding 100,000 mIU/mL in choriocarcinoma, and typically lower values observed in PSTT and ETT. However, normalization of β-hCG levels following a molar pregnancy does not completely exclude the possibility of malignant transformation. In this context, Descargues et al. conducted a retrospective observational study on 7761 patients treated for gestational trophoblastic disease between 1999 and 2020, all of whom experienced spontaneous normalization of human chorionic gonadotropin levels. Among these, 20 patients (0.26%) developed gestational trophoblastic neoplasia despite initial remission [57].

Furthermore, imaging plays a key role in the diagnostic work-up of GTN: pelvic ultrasound is typically used for the initial assessment, while chest X-rays and CT/MRI are crucial for detecting metastatic spread.

GTN is staged anatomically using the 2000 FIGO staging system and is scored prognostically with the 2021 modified WHO prognostic index score, which stratifies patients into low-risk (score ≤ 6) and high-risk (score ≥ 7) categories and comprises age, antecedent pregnancy, interval from index pregnancy (in months), pre-treatment hCG (in mIU/mL), largest tumor size including uterus (in cm), and site of metastases including uterus, number of metastases identified, and previous failed chemotherapy. The 2021 modified WHO prognostic index score is summarized in Table 1.

Because PSTT and ETT are relatively chemoresistant, surgical resection, typically via hysterectomy, is the preferred first-line treatment in non-metastatic disease. In metastatic or recurrent cases, chemotherapy (e.g., EMA/EP or TP/TE regimens) may be considered, though outcomes are poorer compared to other GTNs. The survival rate is approximately 100% for non-metastatic disease and 50–60% for metastatic disease [59].

### 3.4. Management in GTD and New Approaches: Immunotherapy

Because gestational trophoblastic disease is uncommon, there is little evidence from randomized controlled trials indicating the most effective treatment and appropriate follow-up modalities. Treatment protocols differ among European countries and even among different centers within the same countries. One of the aims of the European Organization for the Treatment of Trophoblastic Diseases (EOTTD) is to standardize treatment practices across Europe [60].

Treatment of gestational trophoblastic disease, according to the guidelines, is based on the severity and type of disease [61].

The classification helps to decide on the most appropriate treatment.

All cases of gestational trophoblastic disease, in case of a high-risk situation, should be discussed at a multidisciplinary cancer case conference and registered in a centralized (regional and/or national) database.

Before treating gestational trophoblastic disease, the following is assigned:

- A stage based on the 2000 International Federation of Gynecology and Obstetrics (FIGO) staging system;
- A risk score based on the World Health Organization (WHO) modified prognostic scoring system.

Both systems correlate with clinical outcomes and identify patients at risk of treatment failure [62].

A score between 0 and 6 suggests a low risk that the disease may develop resistance to chemotherapy with a single agent, such as methotrexate with folinic acid (MTX/FA) or actinomycin D (ActD). For women who have already completed their family and have no metastases, the option of hysterectomy may be considered [63].

When a hydatidiform mole is suspected through ultrasound (Doppler), it is important to measure hCG levels, determine blood type, and perform cross-testing and request blood availability.

Uterine vacuum aspiration is the procedure of choice for the uterine evacuation of patients with molar pregnancy, because it is safe and effective. It can be performed by electrical aspiration or by intrauterine manual aspiration, under ultrasound guidance, making sure that blood units are immediately available as bleeding may occur during the procedure [64].

Although there is no evidence that ultrasound guidance leads to more complete evacuations or a lower risk of perforation, this technique is generally accepted and in line with recommendations in the literature on the treatment of gestational trophoblastic disease (GTD) [65].

The diagnosis should be confirmed by histologic examination, possibly supported by additional techniques such as genotyping and p57kip2 staining [66].

Monitoring hCG after evacuation is essential to detect any development of GTN (gestational trophoblastic neoplasia) after a molar abortion. It is advisable to employ a test capable of detecting all variants of hCG (such as beta-hCG, core hCG, C-terminal hCG, nickless beta, beta-core, and hyperglycosylated) uniformly, often referred to as the “total hCG test”. Measurement of hCG is generally conducted regularly every week until levels normalize or GTN is confirmed. However, the optimal duration of hCG monitoring can vary considerably depending on the histologic subtype and the policies of the institution involved in surveillance.

For selected patients a further suction curettage may also prevent the need for chemotherapy, but where the hCG is greater than 5000 IU/L and/or the disease is within the uterine wall rather than the cavity, repeated curettage is likely to fail and is not recommended [67].

If metastases are suspected, diagnostic imaging examinations may be performed. If available, genetic analysis may be considered if deemed necessary. In cases of patients with a negative Rhesus group, it is important to evaluate anti-D prophylaxis.

Histologic confirmation of GTD is mandatory after evacuation. Once the diagnosis of partial or complete mola is conferred, monitoring with hCG measurements at least every two weeks should be initiated. Partial mola do not require prolonged follow-up and can be discontinued once hCG levels have normalized. For complete grinding wheels, because the risk of recurrence even after normal hCG is higher, continued monitoring up to 6 months is recommended.

Although standard therapies, such as chemotherapy, are very effective, there are cases when the disease does not respond or recurs, making alternative approaches necessary.

Over the past decade, immune checkpoint-based immunotherapy (IPC), which uses drugs targeting inhibitory T-cell receptors, such as programed cell death 1 (PD-1) and its ligand (PD-L1), as well as cytotoxic T-lymphocyte-associated antigen 4, has revolutionized therapies for several cancers, including melanoma, non-small cell lung cancer, and renal cell carcinoma.

While the details of the mechanisms of action remain under study, IPCs generally enhance the ability of T cells to respond effectively, thereby enhancing the immune response against malignancies [68].

In the tumor microenvironment, PD-1 binds to PD-L1 and activates its pathway, thereby inhibiting the activity of T lymphocytes. Once the activity of T lymphocyte is inhibited, it cannot kill tumor cells, which survive by immune escape. The use of PD-1 or PD-L1 inhibitors can block the PD-1/PD-L1 pathway, thereby enhancing the activity of T lymphocytes, enhancing the recognition and killing effect of tumor cell [69]. Placental antigen expression in pregnant women makes it a target for immune recognition during pregnancy, while PD-L1 expression maintains pregnancy tolerance. Studies have shown that PD-L1 is expressed in tumor tissues of GTN patients [70,71].

Immunotherapy with the anti-program death-1 (anti-PD-1) antibody can treat patients with gestational trophoblastic neoplasia who are resistant to the usual chemotherapeutics and offers a powerful new treatment with low toxicity.

This development should prompt a rethinking of how patients with this rare disease are managed, focusing on maximizing the cure rate with minimal exposure to toxic chemotherapy [72].

In GTN, studies have focused on the use of agents that block PD-1 (such as pembrolizumab, toripalimab, camrelizumab), or PD-L1 which is normally expressed by tumor cells and immune cells (such as avelumab) [73].

Of course, immunotherapy can lead to adverse events, especially in terms of fertility. This is because IPCs can affect the endocrine system leading to knock-on consequences on the reproductive system.

Similarly, by activating the patient’s immune system with immunotherapy, a broad range of immune-related adverse events can occur that could negatively affect the fetus or impede a future desired pregnancy.

Careful evaluation of long-term fertility outcomes in particular is required since treatment with immune checkpoint inhibitors enhances immune responses against trophoblast cells.

Unfortunately, there are currently still few data available to be able to confirm these long-term adverse effects [74,75].

Although data are still limited and mainly from clinical cases or small studies, the promising results suggest that immunotherapy may be a future strategy to treat forms of MTG that are particularly aggressive or resistant to conventional therapies. However, further studies are needed to evaluate the safety, efficacy, and mode of use of these therapies in this setting.

Studies involved in GTN management are summarized in Table 2.

### 3.5. Fertility and Reproductive Outcomes After GTD

It is very important to elucidate the aspect of future fertility and reproductive outcomes after gestational trophoblastic disease (GTD), considering both a hydatidiform mole but also low-, high-, and ultralight-risk neoplastic trophoblastic disease (GTN), as well as the very rare placental site trophoblastic tumors (PSTT) and epithelioid trophoblastic tumors (ETT).

It is interesting to understand in depth the impact of different types of treatment (single-agent, multi-agent, high-dose chemotherapy, immunotherapy) on fertility, pregnancy desire, and obstetric outcomes [77].

In the case of hydatidiform mola and low-risk GTN, single-drug chemotherapy does not negatively impact fertility or carrying subsequent pregnancies to term; however, conception within 6 months after the end of chemotherapy results in a higher rate of miscarriage [76].

Matsui et al. conducted a study of 137 women with GTD and showed a significantly higher risk of miscarriage (37.5%) if pregnancy occurred within 6 months of chemotherapy, compared with 10.5% t for those expecting beyond 12 months. [78]

Regarding the risk of subsequent molar events after trophoblastic disease, Eagles conducted the most extensive research on the subject. The study examined 16,000 women with both complete and partial hydatidiform molar diagnoses registered at a referral center over 20 years. The results revealed that the risk of developing a new molar pregnancy for women who had previously had a complete hydatidiform mole was 1 in 100. This risk increased significantly, to 1 in 4, in women who had had one or two consecutive complete moles. In contrast, women with partial moles had only a slightly increased risk of recurrence [79].

Polychemotherapy can result in an anticipation of menopausal age by about three years and induce transient amenorrhea; however, in young patients, permanent ovarian failure is a rare event. High-dose chemotherapy results in permanent amenorrhea. No cases of pregnancy after this treatment are described in the literature. As for immunotherapy, it represents a promising new pharmacological approach that nevertheless needs further investigation to clarify its impact on fertility; however, pregnancies have been reported after this form of treatment. In women with GTD, therefore, future fertility is quite safe. Clearly, the most impactful factors on the possibility of having offspring in such a case are age and the type of treatment given for GTD [80].

Particularly in cases of high-dose chemotherapy, after which no cases of spontaneous pregnancy have been reported in the literature, fertility preservation methods can be considered. One of the most recommended fertility preservation methods is oocyte or embryo freezing: these protocols require a couple of weeks to perform proper ovarian stimulation, followed by oocyte retrieval. The disadvantage of this approach is clearly the longer waiting times, so it is recommended for women who can afford a delay before starting gonadotoxic therapy [81].

Another strategy for preserving the fertility of these women is ovarian tissue cryopreservation. Its advantage, compared to gamete or embryo cryopreservation, is its speed: it can be performed within a few days, typically within 6 days of counseling with the IVF team [82].

### 3.6. Complete Familiar Hydatidiform Mole: A Particular Variant

A rare form of molar pregnancy is known as the familial complete hydatidiform mole. It is defined as such due to its recurrence among blood relatives and its autosomal recessive inheritance pattern. This variant is caused by abnormal trophoblastic proliferation in the absence of recognizable embryonic structures. It differs from sporadic variants in its genetic etiology, as evidenced by studies conducted on families with multiple documented cases of complete mole. The gene most certainly involved in the etiology of this variant is NLRP7. The KHDC3L gene may also be involved, albeit more rarely. In all cases, these are maternal genes involved in oocyte epigenetic regulation. Consequently, this variant of trophoblastic disease is not due, as in sporadic forms, to defects in fertilization, but rather to an intrinsic defect in the oocyte [17].

In a recent study, 113 patients with a recurrent hydatidiform mole were observed in order to identify the underlying genetic causes, describe the impact of mutations in known genes, and improve the diagnostic approach and genetic counseling. The patients studied had a history of at least two episodes of a hydatidiform mole, and the impact of the NLRP7 and KHDC3L genes was clarified. It was found that 75% of patients with recurrent molar pregnancy have biallelic mutations either in the NLRP7 gene, which is the most common variant, or in the KHDC3L gene, which is a more unusual but also more severe variant. Patients with these mutations have exclusively molar or abortive pregnancies, with no physiological pregnancies in most cases. In some rare cases, normal pregnancies may occur, but with very low frequency. Targeted sequencing is essential for diagnosis: it is recommended to test the NLRP7 and KHDC3L genes in patients with at least two episodes of complete moles. The diagnosis of this familial variant has aspects in common with sporadic forms; in fact, the key role of transvaginal ultrasound and β-hCG remains unchanged. In addition, genetic analysis of molar tissue and genetic testing of the patient to identify the mutations involved are required for a correct diagnosis [83].

### 3.7. Main Histological Features of GTD Variants

A brief overview of the main characteristics and histological differences between the various variants of GTD is possible. A complete hydatidiform mole is characterized histologically by enlarged, hydropeic chorionic villi with typical central edema; it is also distinguished by cytological atypia, numerous mitoses, and the absence of embryonic tissue. In a partial hydatidiform mole, we find the presence of mixed chorionic villi, some with edema and others that are normal or fibrotic; we also distinguish trophoblast as having focal proliferation and the presence of embryonic or fetal tissue. An invasive mole results from a complete mole that invades the myometrium or uterine vessels, but retains the presence of chorionic villi in the infiltrating tissue. Despite the presence of villi, it is defined as a malignant form due to its infiltrative and metastatic tendency. Choriocarcinoma, also considered a malignant and metastatic form, is characterized by the absence of chorionic villi. The tissue is characterized by a component of anaplastic trophoblastic cells, atypia, necrosis, and hemorrhage. A placental site tumor is defined histologically by the proliferation of mononuclear trophoblastic cells, with irregular nuclei infiltrating the myometrium. The epithelioid trophoblastic tumor is characterized by uniform, mononuclear cells with eosinophilic or clear cytoplasm, arranged in nodules or cords; a distinctive feature is the often extensive necrosis in this histological variety, which is accompanied by possible calcifications [84].

## 4. Conclusions

In recent years, the clinical management of gestational trophoblastic diseases (GTD) has benefited from significant advances in diagnostic, prognostic, and therapeutic approaches. The introduction of increasingly sensitive imaging techniques, together with serial monitoring of serum β-hCG levels, now enables the early detection of initial forms of a hydatidiform mole and gestational trophoblastic neoplasia (GTN), thereby reducing the incidence of complications and metastases. Staging according to the international FIGO criteria, integrated with the WHO prognostic scoring system, is an essential standard for risk stratification and for identifying the most appropriate therapeutic regimen, distinguishing patients into low- and high-risk groups.

Chemotherapy, the cornerstone of GTN treatment, achieves response rates exceeding 95% in low-risk cases thanks to single-agent protocols based on methotrexate or actinomycin D. In high-risk cases, multi-agent regimens—particularly the EMA/CO protocol—have ensured very high rates of complete remission and overall survival, albeit with non-negligible hematologic and long-term toxicities. The emergence of drug resistance, as well as the management of relapses and chemoresistant forms, remains an area of active research, with immunotherapy and targeted therapies representing promising frontiers for clinical development.

A growing area of concern is fertility preservation and reproductive health management during long-term follow-up. Available data indicate that the majority of patients undergoing conservative treatments or low-toxicity chemotherapy regimens maintain a favorable reproductive prognosis, although conception is generally advised to be postponed for at least 6–12 months after serum marker normalization, in order to minimize the risk of recurrence and obstetric complications. A multidisciplinary approach—integrating gynecologic oncologists, pathologists, radiologists, and psycho-oncological support—remains essential to ensure a safe and personalized care pathway based on the latest evidence.

In conclusion, the literature review demonstrates that appropriate diagnostic assessment, accurate staging, and risk-adapted therapeutic strategies, together with structured and prolonged follow-up, are crucial for optimizing oncological and reproductive outcomes in patients with GTD. Nevertheless, significant challenges remain, particularly concerning resistant or recurrent neoplastic forms, which require further clinical and translational research efforts to develop increasingly effective and less toxic innovative approaches.

## Figures and Tables

**Table 1:** This classification is crucial for guiding treatment. Low-risk GTN is typically managed with single-agent chemotherapy, most commonly methotrexate or actinomycin-D, with success rates exceeding 95% [58]. High-risk GTN, including metastatic choriocarcinoma, is treated with multi-agent chemotherapy (EMA/CO), achieving long-term survival rates of 85–94% and a complete response rate of 71–78%. In cases that are resistant to the EMA/CO regimen, the most commonly used alternative is the EMA/EP protocol, in which cyclophosphamide and vincristine are replaced with etoposide and cisplatin.

Risk Factor	0	1	2	4
Age (years)	<40	≥40	-	-
Antecedent pregnancy	Mole	Abortion	Term	-
Interval (months)	4	4 to 6	7 to 12	>12
Pretreatment serum hCG (mIU/mL)	<10^3^	10^3^ to 10^4^	10^4^ to 10^5^	>10^5^
Largest tumor (including uterus)	<3 cm	3 to 4 cm	≥5 cm	-
Site of metastases	Lung	Spleen, kidney	Gl tract	Brain, liver
Number of metastases	-	1 to 4	5 to 8	>8
Prior failed chemotherapy	-	-	Single drug	≥2

**Table 2:** GTN Management.

Study	Patient Number	Diagnosis	Treatment	Outcome
[39]	150	Low-risk post-molar GTN	MTX	a high β-hCG value one week after the first course of MTX predicts the need for additional courses or a change in therapy
[43]	35 studies	High-risk GTN	EMA-CO	complete response rate of 72–91% and overall survival of up to 94% in some cohorts
[44]	30	High-risk GTN with metastases	EMA-CO	93% survival and 67% permanent clinical response
[64]	4	Multi-drug resistant GTN	Pembrolizumab	3 complete remissions
[66]	20	High-risk chemo-resistant GTN	Camrelizumab plus apatinib	10 complete responses
[65]	4	Multi-drug resistant GTN	Toripalimab	all achieved complete remission and the treatment was tolerable
[67]	7	High-risk Multi-drug resistant GTN	Avelumab	1 of 7 remissions
[76]	66	Multidrug resistant disease	Camrelizumab –Sintilimab –Toripalimab – Pembrolzumab	45 of 66 complete remissions
